# *In vitro *evaluation of photon and carbon ion radiotherapy in combination with chemotherapy in glioblastoma cells

**DOI:** 10.1186/1748-717X-7-9

**Published:** 2012-01-27

**Authors:** Stephanie E Combs, Lisa Zipp, Stefan Rieken, Daniel Habermehl, Stefan Brons, Marcus Winter, Thomas Haberer, Jürgen Debus, Klaus-Josef Weber

**Affiliations:** 1 Department of Radiation Oncology, University of Heidelberg, Im Neuenheimer Feld 400, 69120 Heidelberg, Germany; 2 Heidelberg Ion Therapy Center (HIT), Im Neuenheimer Feld 450, 69120 Heidelberg, Germany

**Keywords:** Human glioma cells, carbon ion radiotherapy, chemotherapy, clonogenic survival

## Abstract

**Background:**

To evaluate the cytotoxic effect of carbon ion radiotherapy and chemotherapy in glioblastoma cells *in vitro*.

**Methods and Materials:**

The human glioblastoma (GBM) cell line U87 was irradiated with photon radiotherapy (RT) doses of 2 Gy, 4 Gy and 6 Gy. Likewise, irradiation with carbon ions was performed with single carbon doses of 0.125, 0.5, 2 and 3 Gy. Four chemotherapeutic substances, camptothecin, gemcitabine, paclitaxel and cisplatinum, were used for single and combination experiments. The assessment of the effect of single and double treatment on cell viability was performed using the clonogenic growth assay representing the radiobiological gold standard.

**Results:**

The RBE of carbon ions ranges between 3.3 and 3.9 depending on survival level and dose. All chemotherapeutic substances showed a clear does-response relationhips. in their characteristic concentrations. For subsequent combination experiments, two dose levels leading to low and medium reduction of cell survival were chosen. Combination experiments showed additive effects independently of the drugs' mechanisms of action. Paclitaxel and campthothecin demonstrated the most prominent cytotoxic effect in combination with carbon ion radiotherapy.

**Conclusion:**

In conclusion, combination of carbon ion radiotherapy with chemotherapies of different mechanisms of action demonstrates additive effects. The most dominant effect was produced by paclitaxel, followed by camptothecin, as espected from previously published work. The present data serve as an important radiobiological basis for further combination experiments, as well as clinical studies on combination treatments.

## Background

Novel radiotherapeutic treatment approaches for patients with glioblastoma (GBM) may enable the radiation oncologist to increase local control, and thus impact on progression-free survival and overall survival times; this includes the application of novel radiation qualities, technical advances, dose and fractionation concepts, as well as combined treatment modalities. In spite of extensive research, current outcome after the standard treatment, consisting of postoperative photon radiotherapy in combination with the alkylating substance temozolomide (TMZ) is around 15 months [1].

Particle radiotherapy, such as proton or carbon ion radiation, offers distinct physical characteristics leading to a more conformal dose distribution: Due to the inverted dose profile with low dose deposition in the entry channel of the beam, and high local doses in the so called Bragg Peak, normal tissue surrounding the tumor area can be spared, and the integral dose to the patient can be reduced. Additionally, carbon ions, offer a higher relative biological effeciveness (RBE) due to the severe radiation damage produced within the beam track [2-4]. Most likely the extent of cell death depends on difficult-to-repair double-strand breaks of the DNA [5,6].

Several *in vitro *studies including our own work have shown that, for GBM, the RBE of carbon ions is between 3 and 5, depending on the cell line and the endpoint [7-9]. Comparable to radiochemotherapy with protons, we could show that combination of carbon ions and TMZ lead to an additive effect with respect to cytotoxicity [7].

Several studies have evaluated the combination of chemotherapy with radiation using X-rays, however, only few data is available on the effect of chemotherapy and carbon ion radiotherapy. It has been hypothesized that, due to the different radiobiological effects of high-LET particle beams with special respect to impact on cell cycle control, combination effects known from photon radiotherapy in combination with chemotherapeutic substances of different working mechanisms might be different for carbon ions. A study by Kitabayashi and colleagues evaluated carbon ion radiotherapy and different chemotherapies in esophagenal cell lines, showing that combination with docetaxel was the strongest of 4 combinations revealing promising combination effects [10]. However, for each cancer type distinct groups of chemotherapy have been shown to be effective, therefore it may not hold true to transfer such results to cancer cells in general. Since particle therapy seems a promising treatment alternative for high-grade primary brain tumors, the focus of the present analysis was the evaluation of radiochemotherapy with carbon ions in combination with several chemotherapeutic drugs in glioma cells.

## Materials and methods

### Reagents and Cell Culture

The human glioblastoma (GBM) cell line U87 was obtained from the American Type Culture Collection (ATCC, Manassas, VA, USA). The cells were cultured in DMEM supplemented with 10% fetal calf serum (FCS); they were maintained in culture at 37°C with 5% CO_2 _and 95% humidity.

### Radiotherapy (RT) with photons

Cells were irradiated at room temperature. RT was performed as single exposure to doses of photon RT of 2 Gy, 4 Gy and 6 Gy delivered by a linear accelerator (Fa. Siemens, Erlangen, Germany).

### Radiotherapy (RT) with carbon ions

Irradiation with carbon ion was performed at the Heidelberg ion Therapy Center (HIT) with the horizontal beamline using the rasterscanning technique. To treat cell cultures with clinically relevant parameters we delivered the dose as an extended Bragg peak with 103 keV/μm (dose averaged LET). The position of the Breagg peak was adjusted using a 1 cm acrylic shield, and cell monolyers were placed in the middle ot the extended Bragg peak. Single carbon doses of 0.125, 0.5, 2 and 3 Gy were applied.

### Chemotherapeutic Treatment

U87 cells were treated with chemotherapy at various concentrations 4 hours prior to radiation treatment followed by a medium change to discontinue drug exposition. Four chemotherapeutic substances, camptothecin, gemcitabine, paclitaxel and cisplatinum, were used for single and combination experiments using differenct substance-specific concentrations.

For all substances, dose-response-relationships were generated for single-agent treatment. For combination experiments, low, i.e. ~70-80% survival, and medium, i.e. ~ 50-60% cell survival, toxicity concentrations were chosen: For paclitaxel, 5 nM and 20 nM, 10 nM and 30 nM for gemcitabine, 0.5 μM and 1 μM for cisplatinum, and 10 nM and 30 nM for camptothecin.

For combination experiments, glioma cells were irradiated immediately after drug wash-out by medium change.

### Clonogenic Assay

The assessment of the effect of single and double treatment on cell viability was performed using the clonogenic growth assay representing the radiobiological gold standard. Clonogenic survival is an important criterion of cell survival in repsonse to antitumor agents because the final cell death may occur only after additional cell divisions. For these analyses, the GBM cell lines were grown under standard conditions. To investigate the effect of photon and carbon ion irradiation alone, chemotherapy alone, or the combination of both, on clonogenic survival, increasing numbers of cells (10^2 ^to 5 × 10^4^) were plated in 25 cm^2 ^flasks (Becton Dickinson, Heidelberg, Germany) as described previously [11-13]. Cells were kept in the incubator for an attachment period, and were exposed to compounds and cultures in various concentrations and returned to the incubator for 10-14 days after which they were stained with crystal violet (Sigma-Aldrich, Munich, Germany). Colonies were counted by microscopic inspection and plating efficiency as well as clonogenic survival were calculated. Only colonies with a minimum of 50 cells were counted. Plating efficiencies ranged from 4% to 12% for separate culture preparations. Each experiment was repeated on three separate days, and on each day triplicates of each dose and treatment combination was performed. Mean values (and standard deviations) were only calculated from independent experiments (separate days).

## Results

### Clonogenic survival and determination of RBE

**Figure 1:**
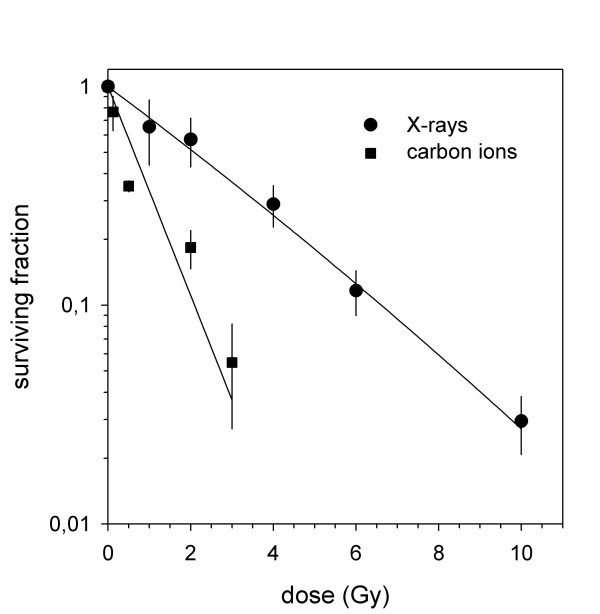
**Clonogenic survival after photon and carbon ion radiotherapy**. Carbon ion radiotherapy reveals a stronger cytotoxic effect in glioblastoma cells compared to photon radiotherapy.

**Figure 2:**
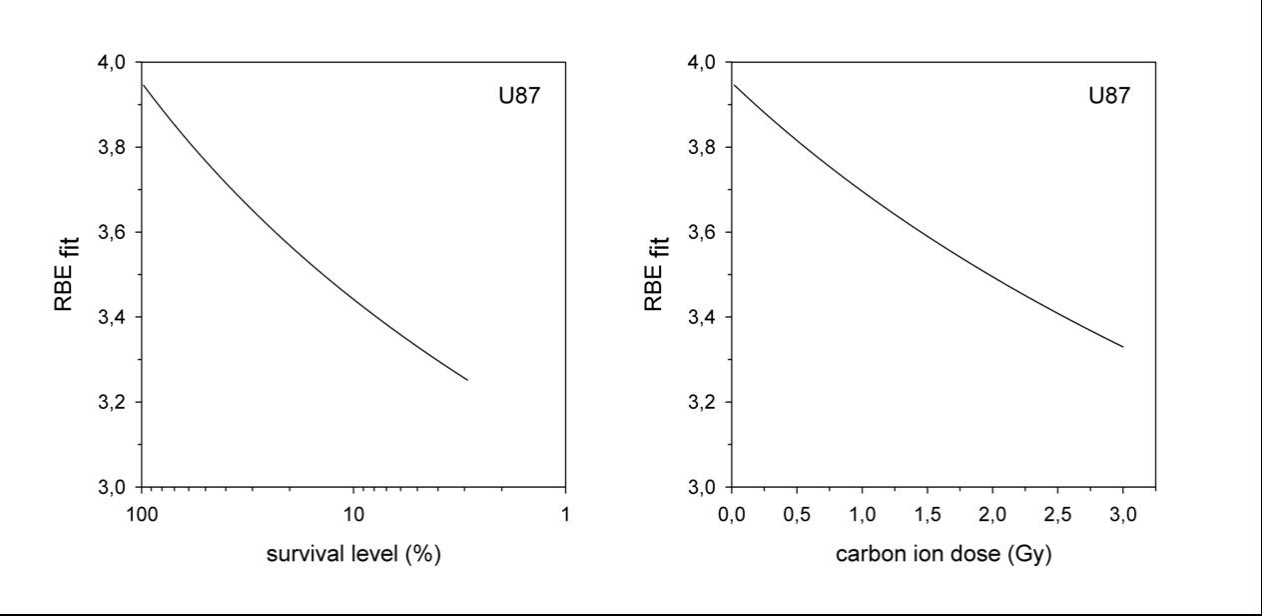
**Relative biological effectiveness (calculated from LQ-fits) as a function of survival level (A) or carbon ion dose (B)**. The RBE ranges between 3.3 and 3.9 depending on survival level and dose.

We calculated clonogenic survival after increasing doses of photon and carbon ion radiotherapy on U87 cells; carbon ion radiotherapy demonstrated a steeper dose-response-relationship revealing a stronger cytotoxic effect in glioblastoma cells compared to photon radiotherapy (Figure 1). From these values, we calculated the RBE from linear-quadratic-fits (LQ-fits) as a function of survival level (Figure 2A) or carbon ion dose (Figure 2B). The RBE ranges between 3.3 and 3.9 depending on survival level and dose.

#### Clonogenic survival after single-agent chemothrapies

**Figure 3:**
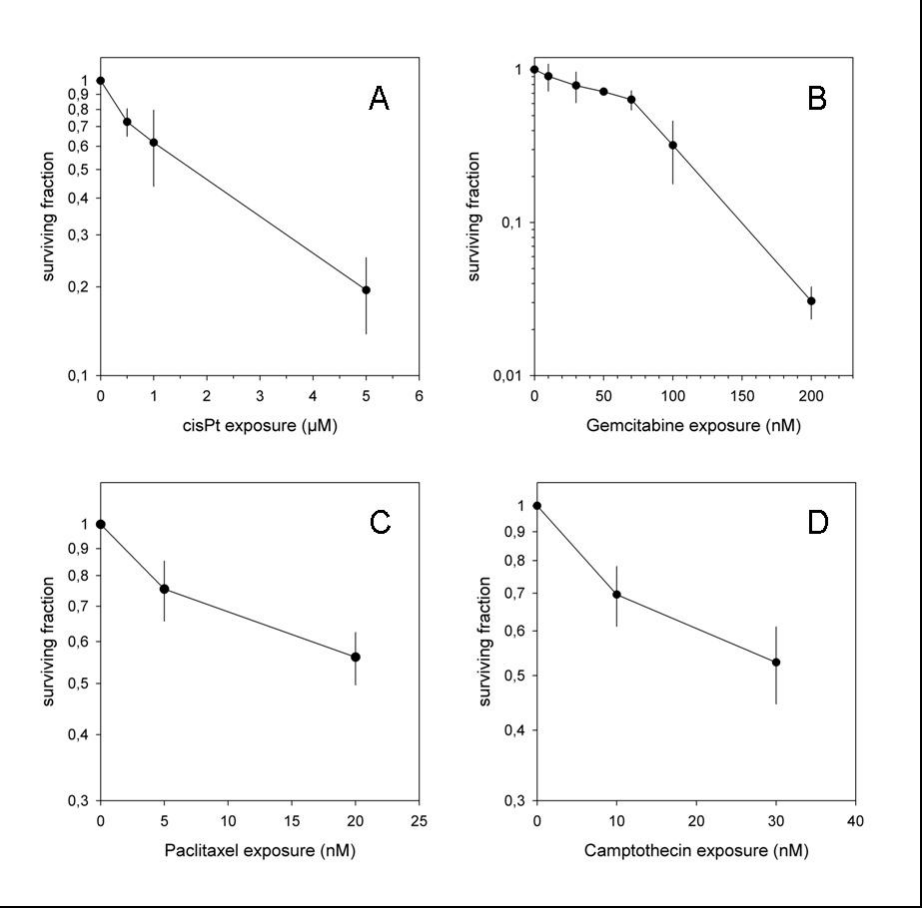
**Clonogenic survival of U87 cells treated with increasing doses of cisplatinum (A), camptothecin (B), gemcitabine (C) and paclitaxel (D)**.

The *in vitro *survival curves of U87 treated with increasing concentrations of gemcitabine, paclitaxel, camptothecin and cisplatinum are shown in Figure 3. All substances showed a clear does-response relationship in their characteristic concentrations. For subsequent combination experiments, two dose levels leading to low and medium reduction of cell survival were chosen.

### Evaluation of cytotoxic effect of radiation and chemotherapy

**Figure 4:**
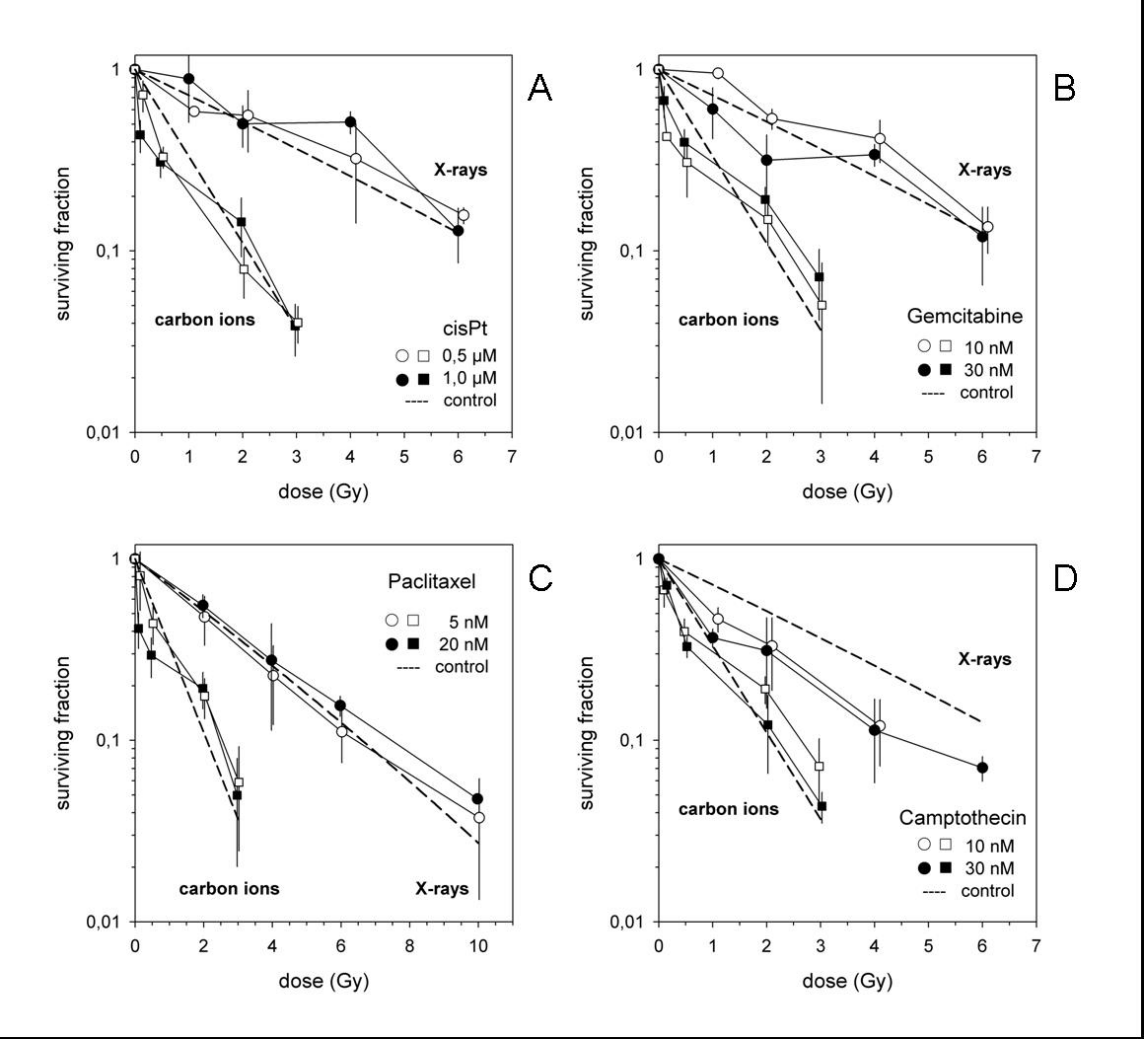
**Combination experiments of 4 chemotherapeutic drugs (cisplatinum (A), gemcitabine (B), paclitaxel (C) and camptothecin (D)**. Results showed additive effects independently of the drugs' mechanisms of action.

Combination experiments of 4 chemotherapeutic drugs cisplatinum, gemcitabine, paclitaxel and camptothecin (Figure 4 A-D). For generation of the survival curves within each independent experiment, PE-values measured in triplicate for each treatment condition and the averaged values were normalized to the respective number for the untreated sample. Respective PE-values obtained in the combination experiments were normalized to a drug control yielding surviving fractions diplayed in the graphical representation. Results showed additive effects independently of the drugs' mechanisms of action. No agent demonstrated a significantly higher combination effect compared to the other substances.

**Figure 5:**
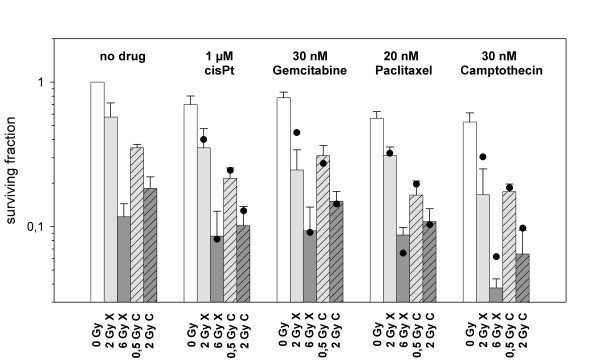
**Bar diagrams of surviving fractions of U87 cells (X X-rays, C Carbon ions, • expected survival according to independent toxicity)**. For all substances additive effects could be observed. In U87, paclitaxel and campthothecin demonstrated the most prominent cytotoxic effect in combination with carbon ion radiotherapy.

Bar diagrams of surviving fractions of U87 cells measured *in vitro *as well as calculated from independent toxicities obtained from single agent experiments (Figure 5). For all substances additive effects could be observed. This holds true also for the more effective agents, paclitacel and camptothecin, resulting in a prominent cytotoxic effect for the combination with carbon ions.

## Discussion

The present study evaluated the combination of different chemotherapeutic substances in combination with photon and carbon ion radiotherapy with respect to cell survival in glioblastoma cells. The work shows additive effects for all drugs independently of their mechanisms of action, however, revealing the strongest interactive effect for paclitaxel and campthothecin.

Until now, few data is available on the combination of chemotherapy and high-LET particle beams, such as carbon ion radiotherapy. From a clinical perspective carbon ion radiotherapy might be a promising treatment alternative in patients with GBM, which has been reported by a Japanese study [14]. The potential additive value of a carbon ion boost is currently evaluated in a randomized clinical trial [15]. This clinical work had been based on a previous experimental study from our group: We could show that combination of temozolomide, an alkylating chemotherapeutic drug, and carbon ion radiotherapy leads to an additive effect, comparable to photons [7].

To further exploit a possible combination of other drugs with alternative mechanisms, we included agents commonly not in clinical use from GBM into the present work to identify the combination effect with high-LET carbon ion radiotherapy. Four different chemotherapeutics were used, and paclitaxel as well as camptothecin showed the strongest additive effects in combination with carbon ions, compared to gemcitabine and cisplatinum. These results are in line with a previous study published by Kitabayashi et al., evaluating different chemotherapeutic substances in esophageal squamous cell carcinoma lines [10]. The authors could show that docetaxel and heavy ion radiotherapy lead to the most prominent reduction of cell survival *in vitro *and *in vivo*.

Novel treatment approaches such as molecularly-targeted subtances, might potentiate the effect of carbon ion radiotherapy, and preclinical work is currently ongoing. Only recently, Kano and collegues evaluated histone-deacetylase inhibitors in combination with carbon ions in esophageal cell lines [16]; a radiosensitizing effect could be observed, and tumor growth was significantly suppressed by the combination of carbon ion radiotherapy with the histone deacetylase inhibitor in comparison to either agent alone. Similar data have been reported for sarcoma cell lines using a combination of a pan-HDAC inhibitor (HDACI) suberoylanilide hydroxamic acid (SAHA) in combination with carbon ion radiotherapy [17]. The medication lead to an increase of sensitivity to carbon ion radiotherapy along with an increase of double strang breaks and apoptosis.

In the clinical setting, particle therapy has to be included into existing treatment protocols, which in most cases include radiation and chemotherapy. Therefore, when introducing a new radiation quality, the effect of combined treatment is essential information to calculated risks of side effects, and therapeutic ratios. This manuscript presents the first extensive evaluation of the combination effect of carbon ions and chemotherapeutic substances of different mechanisms of action, focussing on GBM cell lines. Therefore the present work represents essential radiobiological information for subsequent pre-clinical as well as clinical applications, leading to the conclusion that when such substances are combined with carbon ions no specific reductions in dosing with respect to cytotoxicity in the radiation area must be undertaken compared to combined treatments with photons. The results also pertain the aspect of biological plan optimization in carbon ion radiotherapy: Biologic plan optimization is used for all treatment plans in clinical practice; in Heidelberg, the local effect model (LEM) integrating various parameters has been established in clinical routine [3,18,19]. Several clinical and theoretical studies have focussed on radiobiological parameters in GBM, and Jones and colleagues estimated αβ ratio, doubling time and other parameters, as well as equivalent biologial radiation dose to temozolomide chemotherapy [20]. These data may serve as an essential basis for further combination treatments, and the present preclinical results underline that it should hold true that biological treatment planning can be transferred also into the setting of radiochemotherapy without having to adjust the known input parameters for each tumor cell type. In our own previous work, we have confirmed this hypothesis [7].

## Conclusion

In conclusion, combination of carbon ion radiotherapy with chemotherapies of different mechanisms of action demonstrates additive effects. The most dominant effect was produced by paclitaxel, followed by camptothecin, as espected from previously published work. The present data serve as an important radiobiological basis for further combination experiments, as well as clinical studies on combination treatments.
